# 2-(2-Benzyl­oxyphen­yl)-1*H*-benzimid­azole

**DOI:** 10.1107/S1600536808004510

**Published:** 2008-02-20

**Authors:** Gabriel Navarrete-Vázquez, Hermenegilda Moreno-Diaz, Samuel Estrada-Soto, Hugo Tlahuext

**Affiliations:** a Facultad de Farmacia, Universidad Autónoma del Estado de Morelos, Avenida Universidad 1001 Col., Chamilpa, CP 62100, Cuernavaca Mor., Mexico; b Centro de Investigaciones Químicas, Universidad Autónoma del Estado de Morelos, Avenida Universidad 1001 Col., Chamilpa, CP 62100, Cuernavaca Mor., Mexico

## Abstract

The asymmetric unit of the title compound, C_20_H_16_N_2_O, contains two mol­ecules. The dihedral angles between the benzimidazole ring systems and the attached benzene rings are 10.6 (5) and 13.7 (5)°. The conformers are linked by bifurcated three-centre hydrogen bonds, forming chains along the diagonal of the *a* *b* plane. The packing is further stabilized by π–π and C—H⋯π inter­actions.

## Related literature

For general background, see: Desiraju & Steiner (1999 ▶); Lehn (1990 ▶); Saenger (1984 ▶); Wakelin (1986 ▶). For related structures, see: Estrada-Soto *et al.* (2006 ▶); Moreno-Diaz *et al.* (2006 ▶); Navarrete-Vázquez *et al.* (2006 ▶).
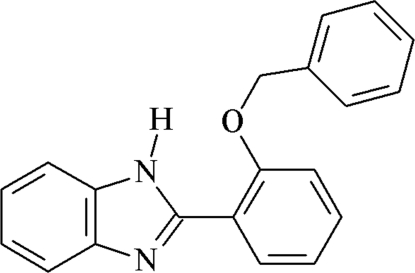


## Experimental

### 

#### Crystal data

C_20_H_16_N_2_O
*M* *_r_* = 300.35
Monoclinic, 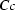

*a* = 11.526 (2) Å
*b* = 17.210 (3) Å
*c* = 15.866 (3) Å
β = 90.52 (3)°
*V* = 3147.0 (11) Å^3^
*Z* = 8
Mo *K*α radiation
μ = 0.08 mm^−1^
*T* = 293 (2) K
0.31 × 0.21 × 0.17 mm

#### Data collection

Bruker SMART CCD area-detector diffractometer
Absorption correction: multi-scan (*SADABS*; Sheldrick, 2003 ▶) *T*_min_ = 0.976, *T*_max_ = 0.987
7808 measured reflections
2830 independent reflections
2691 reflections with *I* > 2σ(*I*)
*R*_int_ = 0.038

#### Refinement

*R*[*F* ^2^ > 2σ(*F* ^2^)] = 0.048
*wR*(*F* ^2^) = 0.100
*S* = 1.17
2830 reflections
423 parameters
2 restraints
H atoms treated by a mixture of independent and constrained refinement
Δρ_max_ = 0.20 e Å^−3^
Δρ_min_ = −0.20 e Å^−3^

### 

Data collection: *SMART* (Bruker, 2000 ▶); cell refinement: *SAINT-Plus NT* (Bruker, 2001 ▶); data reduction: *SAINT-Plus NT*; program(s) used to solve structure: *SHELXTL-NT* (Sheldrick, 2008 ▶); program(s) used to refine structure: *SHELXTL-NT*; molecular graphics: *SHELXTL-NT*; software used to prepare material for publication: *PLATON* (Spek, 2003 ▶).

## Supplementary Material

Crystal structure: contains datablocks I, global. DOI: 10.1107/S1600536808004510/sj2464sup1.cif

Structure factors: contains datablocks I. DOI: 10.1107/S1600536808004510/sj2464Isup2.hkl

Additional supplementary materials:  crystallographic information; 3D view; checkCIF report

## Figures and Tables

**Table 1:** Hydrogen-bond geometry (Å, °) *Cg*1 is the centroid of the C27/N4/C26/C21/N3 imidazole ring, and *Cg*2, *Cg*3 and *Cg*4 are the centroids of the C28–C33, C15–C20 and C35–C40 benzene rings, respectively.

*D*—H⋯*A*	*D*—H	H⋯*A*	*D*⋯*A*	*D*—H⋯*A*
N1—H1⋯O1	0.89 (4)	2.20 (3)	2.667 (4)	112 (3)
N1—H1⋯N4^i^	0.89 (4)	2.18 (3)	3.008 (4)	154 (3)
N3—H3*A*⋯O2	0.83 (4)	2.20 (5)	2.670 (4)	117 (4)
N3—H3*A*⋯N2^ii^	0.83 (4)	2.18 (5)	2.918 (4)	148 (4)
C14—H14*A*⋯*Cg*1^i^	0.97	2.88	3.736 (4)	148
C14—H14*B*⋯*Cg*4^iii^	0.97	2.92	3.721 (4)	141
*Cg*3⋯*Cg*2^i^			3.859 (2)	

Symmetry codes: (i) 
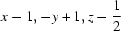
; (ii) 
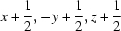
; (iii) 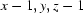.

**Fig. 1.**
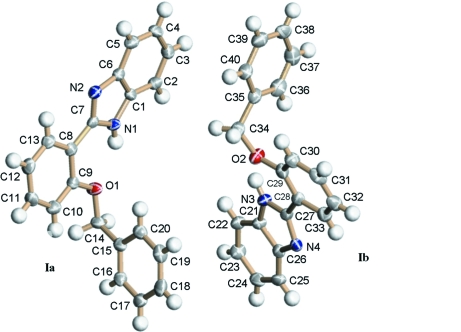
The asymmetric unit of (I) showing the atom-labelling scheme. Displacement ellipsoids are drawn at the 50% probability level and H atoms are shown as small spheres of arbitrary radii.

**Fig. 2.**
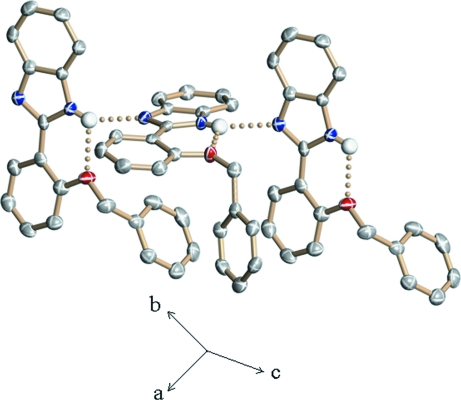
A view of the crystal packing of (I), showing the formation of chains. Hydrogen bonds are represented by dotted lines and H atoms not involved in hydrogen bonding have been omitted for clarity.

**Fig. 3.**
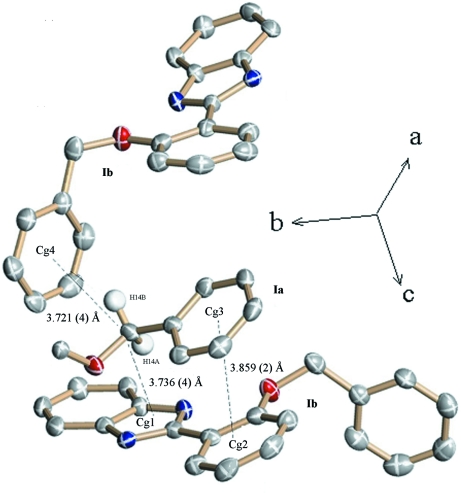
View of the offset π–π interactions and the C–H···π-facial hydrogen bonds between methylene group of Ia and the imidazole and aryl rings of Ib. Dashed lines indicate the vectors between C14 and the benzimidazole and aryl centroids of Ib (Cg1, Cg4). For clarity on part of the Ia molecule is shown.

## supplementary crystallographic information

### Comment

Hydrogen bonding and π–π interactions are among the principal forces which determine the intercalation of drugs into DNA (Saenger, 1984; Wakelin, 1986), together with self-assembly and recognition in some chemical and biological systems (Lehn, 1990). Recently, we have reported the synthesis of a small library of 2-arylbenzimidazole compounds that show spasmolytic and relaxant activity (Moreno-Diaz *et al.*, 2006; Navarrete-Vázquez *et al.*, 2006; Estrada-Soto *et al.*, 2006). In order to extend our research on the relationship between the structure of benzimidazole derivatives and their pharmacological activity we have determined the crystal structure of (I).

The asymmetric unit of (I) contains two conformers (Ia, Ib) (Fig. 1). Bond lengths between equivalent non-H atoms of each conformer are similar, with differences less than 3 s.u. The dihedral angles between the benzimidazole ring systems and the 2-benzyloxyphenyl substituents in Ia and Ib are 10.6 (5) and 13.7 (5)°, respectively. Both molecules display bifurcated N—H···N and N—H···O hydrogen bonds, Table 1 (Desiraju & Steiner, 1999). These hydrogen bonds interconnect molecules into chains running between the *a* and *b* axes (Fig. 2). Packing is further stabilized by C—H···π interactions involving the methylene H atoms on C14 of molecule Ia with the C27N4C26C21N3 benzimidazole ring (centroid *Cg*1) and the C28–C33 benzene ring (centroid *Cg*4) of two adjacent molecules of Ib (Fig. 3, Table 1). In the crystal packing there are also π–π interactions between adjacent molecules Ia and Ib, with a distance between the centroids of the C15–C20 (*Cg*3) and C28–C33 (*Cg*2) benzene rings of 3.859 (2) Å (Fig. 3, Table 1). In conclusion, this crystal structure illustrates four types of cooperative intra and intermolecular interactions: offset π–π stacking and C—H···π interactions as well as classical N—H···N and N—H···O hydrogen bonds.

### Experimental

The title compound, (I), was synthesized according to the method of Moreno-Diaz *et al.* (2006). Single crystals of (I) were obtained from methanol with yield 2.07 g, 75%.

### Refinement

Aromatic and methylene H atoms were positioned geometrically, were constrained to the riding-model approximation [*C*—H_aryl_ = 0.93 Å, *U*_iso_(H_aryl_) = 1.2*U*_eq_(C_aryl_); *C*—H_methylene_ = 0.97 Å, a *U*_iso_(H_methylene_) = 1.5 *U*_eq_(C_methylene_)]. Atoms H1 and H3A, which are involved in hydrogen-bonding interactions, were located in a difference Fourier map and refined freely with isotropic displacement parameters. In the absence of significant anomalous dispersion effects, Friedel pairs were averaged.

### Crystal data

**Table:**

C_20_H_16_N_2_O	*F*_000_ = 1264
*M**_r_* = 300.35	*D*_x_ = 1.268 Mg m^−^^3^
Monoclinic, *C**c*	Melting point: 415.5 K
Hall symbol: C -2yc	Mo *K*α radiation λ = 0.71073 Å
*a* = 11.526 (2) Å	Cell parameters from 68 reflections
*b* = 17.210 (3) Å	θ = 2.6–25.2º
*c* = 15.866 (3) Å	µ = 0.08 mm^−^^1^
β = 90.52 (3)º	*T* = 293 (2) K
*V* = 3147.0 (11) Å^3^	Rectangular, colourless
*Z* = 8	0.31 × 0.21 × 0.17 mm

### Data collection

**Table:**

Bruker SMART CCD area-detector diffractometer	2830 independent reflections
Radiation source: fine-focus sealed tube	2691 reflections with *I* > 2σ(*I*)
Monochromator: graphite	*R*_int_ = 0.038
Detector resolution: 8.3 pixels mm^-1^	θ_max_ = 25.2º
*T* = 293(2) K	θ_min_ = 2.1º
φ and ω scans	*h* = −13→13
Absorption correction: multi-scan(SADABS; Sheldrick, 2003)	*k* = −20→11
*T*_min_ = 0.976, *T*_max_ = 0.987	*l* = −19→16
7808 measured reflections	

### Refinement

**Table:**

Refinement on *F*^2^	Secondary atom site location: difference Fourier map
Least-squares matrix: full	Hydrogen site location: inferred from neighbouring sites
*R*[*F*^2^ > 2σ(*F*^2^)] = 0.048	H atoms treated by a mixture of independent and constrained refinement
*wR*(*F*^2^) = 0.100	*w* = 1/[σ^2^(*F*_o_^2^) + (0.0376*P*)^2^ + 0.4648*P*] where *P* = (*F*_o_^2^ + 2*F*_c_^2^)/3
*S* = 1.17	(Δ/σ)_max_ < 0.001
2830 reflections	Δρ_max_ = 0.20 e Å^−^^3^
423 parameters	Δρ_min_ = −0.20 e Å^−^^3^
2 restraints	Extinction correction: none
Primary atom site location: structure-invariant direct methods	

### Special details

**Table:**

Geometry. All e.s.d.'s (except the e.s.d. in the dihedral angle between two l.s. planes) are estimated using the full covariance matrix. The cell e.s.d.'s are taken into account individually in the estimation of e.s.d.'s in distances, angles and torsion angles; correlations between e.s.d.'s in cell parameters are only used when they are defined by crystal symmetry. An approximate (isotropic) treatment of cell e.s.d.'s is used for estimating e.s.d.'s involving l.s. planes.
Refinement. Refinement of *F*^2^ against ALL reflections. The weighted *R*-factor *wR* and goodness of fit *S* are based on *F*^2^, conventional *R*-factors *R* are based on *F*, with *F* set to zero for negative *F*^2^. The threshold expression of *F*^2^ > σ(*F*^2^) is used only for calculating *R*-factors(gt) *etc*. and is not relevant to the choice of reflections for refinement. *R*-factors based on *F*^2^ are statistically about twice as large as those based on *F*, and *R*- factors based on ALL data will be even larger.

### Fractional atomic coordinates and isotropic or equivalent isotropic displacement parameters (Å^2^)

**Table:**

	*x*	*y*	*z*	*U*_iso_*/*U*_eq_	
C1	0.0469 (3)	0.1160 (2)	0.3249 (2)	0.0179 (8)	
C2	−0.0279 (3)	0.1316 (2)	0.3917 (2)	0.0213 (8)	
H2	−0.0755	0.1752	0.3920	0.026*	
C3	−0.0273 (3)	0.0789 (2)	0.4571 (2)	0.0234 (8)	
H3	−0.0735	0.0880	0.5038	0.028*	
C4	0.0419 (3)	0.0115 (2)	0.4547 (2)	0.0243 (9)	
H4	0.0389	−0.0234	0.4994	0.029*	
C5	0.1134 (3)	−0.0040 (2)	0.3885 (2)	0.0241 (9)	
H5	0.1589	−0.0486	0.3878	0.029*	
C6	0.1160 (3)	0.0492 (2)	0.3217 (2)	0.0176 (8)	
C7	0.1528 (3)	0.1149 (2)	0.2093 (2)	0.0161 (7)	
C8	0.2074 (3)	0.1393 (2)	0.1295 (2)	0.0175 (8)	
C9	0.1951 (3)	0.2138 (2)	0.0950 (2)	0.0183 (8)	
C10	0.2567 (3)	0.2342 (2)	0.0234 (2)	0.0251 (9)	
H10	0.2501	0.2840	0.0012	0.030*	
C11	0.3279 (3)	0.1801 (2)	−0.0149 (2)	0.0250 (9)	
H11	0.3693	0.1943	−0.0625	0.030*	
C12	0.3387 (3)	0.1057 (2)	0.0163 (2)	0.0254 (9)	
H12	0.3854	0.0694	−0.0107	0.031*	
C13	0.2787 (3)	0.0857 (2)	0.0886 (2)	0.0219 (8)	
H13	0.2860	0.0357	0.1103	0.026*	
C14	0.1043 (3)	0.3400 (2)	0.1037 (2)	0.0219 (8)	
H14A	0.0319	0.3604	0.1252	0.026*	
H14B	0.0976	0.3380	0.0427	0.026*	
C15	0.2018 (3)	0.3952 (2)	0.1275 (2)	0.0199 (8)	
C16	0.2041 (3)	0.4669 (2)	0.0885 (2)	0.0229 (8)	
H16	0.1473	0.4796	0.0488	0.027*	
C17	0.2905 (3)	0.5204 (2)	0.1082 (2)	0.0260 (9)	
H17	0.2918	0.5684	0.0813	0.031*	
C18	0.3752 (4)	0.5020 (2)	0.1683 (2)	0.0294 (9)	
H18	0.4334	0.5374	0.1818	0.035*	
C19	0.3714 (3)	0.4304 (2)	0.2071 (2)	0.0276 (9)	
H19	0.4272	0.4180	0.2476	0.033*	
C20	0.2863 (3)	0.3768 (2)	0.1872 (2)	0.0234 (9)	
H20	0.2856	0.3286	0.2136	0.028*	
C21	0.7495 (3)	0.6470 (2)	0.7012 (2)	0.0180 (8)	
C22	0.6466 (3)	0.6399 (2)	0.6541 (2)	0.0224 (8)	
H22	0.6103	0.5922	0.6463	0.027*	
C23	0.6018 (3)	0.7076 (2)	0.6200 (2)	0.0268 (9)	
H23	0.5331	0.7054	0.5889	0.032*	
C24	0.6575 (4)	0.7794 (2)	0.6310 (2)	0.0274 (9)	
H24	0.6257	0.8235	0.6062	0.033*	
C25	0.7588 (3)	0.7861 (2)	0.6781 (2)	0.0237 (9)	
H25	0.7943	0.8340	0.6865	0.028*	
C26	0.8057 (3)	0.7180 (2)	0.7126 (2)	0.0191 (8)	
C27	0.9128 (3)	0.6312 (2)	0.7734 (2)	0.0183 (8)	
C28	1.0124 (3)	0.5952 (2)	0.8170 (2)	0.0196 (8)	
C29	1.0358 (3)	0.5147 (2)	0.8146 (2)	0.0238 (8)	
C30	1.1374 (3)	0.4861 (2)	0.8502 (2)	0.0270 (9)	
H30	1.1533	0.4332	0.8480	0.032*	
C31	1.2149 (3)	0.5359 (3)	0.8887 (2)	0.0301 (10)	
H31	1.2836	0.5164	0.9115	0.036*	
C32	1.1918 (3)	0.6145 (3)	0.8941 (2)	0.0308 (10)	
H32	1.2437	0.6473	0.9217	0.037*	
C33	1.0913 (3)	0.6440 (2)	0.8583 (2)	0.0247 (9)	
H33	1.0761	0.6970	0.8618	0.030*	
C34	0.9791 (4)	0.3883 (2)	0.7630 (2)	0.0288 (9)	
H34A	1.0596	0.3828	0.7468	0.035*	
H34B	0.9314	0.3686	0.7171	0.035*	
C35	0.9579 (3)	0.3394 (2)	0.8407 (2)	0.0232 (8)	
C36	0.8786 (4)	0.3607 (2)	0.9019 (3)	0.0324 (10)	
H36	0.8390	0.4076	0.8970	0.039*	
C37	0.8581 (4)	0.3123 (3)	0.9702 (3)	0.0367 (11)	
H37	0.8049	0.3271	1.0110	0.044*	
C38	0.9165 (4)	0.2422 (3)	0.9780 (3)	0.0320 (10)	
H38	0.9033	0.2102	1.0241	0.038*	
C39	0.9938 (4)	0.2205 (2)	0.9173 (3)	0.0321 (10)	
H39	1.0320	0.1731	0.9221	0.039*	
C40	1.0159 (3)	0.2682 (2)	0.8486 (2)	0.0260 (9)	
H40	1.0689	0.2528	0.8080	0.031*	
N1	0.0711 (3)	0.15646 (17)	0.25172 (18)	0.0174 (6)	
N2	0.1813 (3)	0.04949 (17)	0.24872 (17)	0.0191 (7)	
N3	0.8195 (3)	0.59233 (18)	0.74010 (19)	0.0197 (7)	
N4	0.9075 (3)	0.70755 (17)	0.75867 (17)	0.0180 (7)	
O1	0.1204 (2)	0.26267 (14)	0.13499 (15)	0.0230 (6)	
O2	0.9538 (2)	0.46984 (15)	0.77489 (16)	0.0266 (6)	
H1	0.038 (3)	0.202 (2)	0.241 (2)	0.015 (9)*	
H3A	0.807 (4)	0.545 (3)	0.740 (3)	0.034 (12)*	

### Atomic displacement parameters (Å^2^)

**Table:**

	*U*^11^	*U*^22^	*U*^33^	*U*^12^	*U*^13^	*U*^23^
C1	0.022 (2)	0.0171 (18)	0.0143 (17)	−0.0041 (16)	−0.0013 (14)	0.0012 (14)
C2	0.022 (2)	0.0161 (18)	0.026 (2)	−0.0017 (16)	0.0013 (15)	0.0000 (16)
C3	0.024 (2)	0.027 (2)	0.0197 (18)	−0.0024 (17)	0.0014 (15)	−0.0030 (17)
C4	0.032 (2)	0.023 (2)	0.0186 (18)	−0.0037 (18)	−0.0018 (16)	0.0068 (15)
C5	0.028 (2)	0.0168 (19)	0.027 (2)	−0.0005 (17)	−0.0035 (16)	0.0012 (17)
C6	0.018 (2)	0.0155 (18)	0.0189 (18)	−0.0015 (15)	−0.0006 (15)	−0.0024 (15)
C7	0.0180 (19)	0.0121 (18)	0.0182 (17)	−0.0026 (15)	−0.0024 (14)	−0.0031 (14)
C8	0.0142 (18)	0.0204 (19)	0.0179 (17)	−0.0069 (15)	−0.0038 (14)	−0.0025 (15)
C9	0.021 (2)	0.0167 (18)	0.0170 (18)	0.0002 (16)	−0.0015 (15)	−0.0009 (15)
C10	0.029 (2)	0.020 (2)	0.026 (2)	−0.0009 (18)	−0.0023 (16)	0.0019 (17)
C11	0.027 (2)	0.028 (2)	0.0198 (19)	−0.0064 (18)	0.0087 (16)	−0.0008 (16)
C12	0.028 (2)	0.024 (2)	0.0248 (19)	0.0042 (18)	0.0069 (16)	−0.0085 (17)
C13	0.023 (2)	0.0181 (19)	0.0249 (19)	0.0030 (17)	−0.0031 (15)	−0.0018 (16)
C14	0.027 (2)	0.0172 (19)	0.0217 (19)	0.0021 (17)	0.0031 (15)	0.0033 (16)
C15	0.023 (2)	0.019 (2)	0.0179 (18)	0.0047 (16)	0.0056 (15)	−0.0003 (15)
C16	0.028 (2)	0.0211 (19)	0.0199 (18)	0.0051 (17)	−0.0026 (16)	−0.0024 (16)
C17	0.035 (2)	0.018 (2)	0.026 (2)	0.0010 (18)	0.0052 (17)	−0.0031 (16)
C18	0.031 (2)	0.030 (2)	0.027 (2)	−0.0011 (19)	0.0056 (17)	−0.0133 (18)
C19	0.018 (2)	0.040 (2)	0.025 (2)	0.0062 (18)	−0.0013 (15)	−0.0056 (18)
C20	0.024 (2)	0.025 (2)	0.0206 (19)	0.0045 (17)	0.0041 (16)	0.0008 (16)
C21	0.0165 (19)	0.0150 (17)	0.0225 (18)	−0.0006 (15)	0.0019 (14)	0.0017 (15)
C22	0.022 (2)	0.0182 (19)	0.0270 (19)	−0.0031 (16)	−0.0006 (15)	0.0009 (16)
C23	0.021 (2)	0.028 (2)	0.032 (2)	0.0030 (18)	−0.0043 (16)	0.0006 (18)
C24	0.036 (2)	0.023 (2)	0.024 (2)	0.0075 (18)	0.0045 (17)	0.0053 (17)
C25	0.029 (2)	0.0189 (19)	0.023 (2)	−0.0057 (17)	0.0084 (16)	0.0035 (16)
C26	0.020 (2)	0.0211 (19)	0.0163 (17)	−0.0003 (16)	0.0039 (14)	−0.0010 (15)
C27	0.0184 (19)	0.0175 (18)	0.0191 (17)	−0.0065 (16)	0.0060 (14)	−0.0012 (15)
C28	0.0171 (19)	0.0230 (19)	0.0186 (17)	−0.0029 (17)	0.0068 (14)	−0.0019 (16)
C29	0.026 (2)	0.027 (2)	0.0177 (18)	0.0016 (18)	0.0014 (15)	−0.0022 (17)
C30	0.027 (2)	0.029 (2)	0.0249 (19)	0.0037 (18)	−0.0020 (16)	0.0024 (18)
C31	0.018 (2)	0.048 (3)	0.024 (2)	0.001 (2)	−0.0043 (16)	0.012 (2)
C32	0.027 (2)	0.041 (3)	0.024 (2)	−0.0114 (19)	−0.0042 (17)	0.0071 (19)
C33	0.022 (2)	0.029 (2)	0.0226 (18)	−0.0045 (18)	0.0018 (16)	0.0004 (17)
C34	0.032 (2)	0.023 (2)	0.031 (2)	0.0075 (19)	−0.0077 (18)	−0.0064 (17)
C35	0.020 (2)	0.024 (2)	0.026 (2)	−0.0005 (16)	−0.0070 (16)	−0.0040 (16)
C36	0.024 (2)	0.030 (2)	0.044 (2)	0.0056 (19)	−0.0039 (19)	−0.007 (2)
C37	0.023 (2)	0.053 (3)	0.034 (2)	−0.014 (2)	0.0047 (18)	−0.009 (2)
C38	0.033 (3)	0.029 (2)	0.034 (2)	−0.016 (2)	−0.0012 (19)	0.0032 (19)
C39	0.033 (2)	0.023 (2)	0.040 (2)	−0.0030 (19)	−0.0056 (19)	−0.0005 (19)
C40	0.025 (2)	0.025 (2)	0.029 (2)	0.0021 (18)	0.0007 (16)	−0.0053 (18)
N1	0.0187 (17)	0.0113 (15)	0.0222 (16)	0.0033 (13)	0.0026 (12)	0.0008 (13)
N2	0.0202 (17)	0.0152 (15)	0.0219 (16)	0.0006 (13)	−0.0023 (13)	−0.0016 (13)
N3	0.0213 (17)	0.0133 (16)	0.0244 (16)	−0.0015 (14)	−0.0008 (13)	0.0004 (14)
N4	0.0190 (16)	0.0200 (15)	0.0149 (15)	−0.0029 (14)	0.0011 (12)	−0.0004 (12)
O1	0.0251 (14)	0.0162 (13)	0.0279 (14)	0.0043 (12)	0.0073 (11)	0.0033 (11)
O2	0.0246 (15)	0.0203 (14)	0.0346 (15)	0.0045 (12)	−0.0103 (12)	−0.0028 (12)

### Geometric parameters (Å, °)

**Table:**

C1—N1	1.384 (4)	C21—C26	1.394 (5)
C1—C2	1.399 (5)	C21—C22	1.401 (5)
C1—C6	1.400 (5)	C22—C23	1.382 (5)
C2—C3	1.378 (5)	C22—H22	0.9300
C2—H2	0.9300	C23—C24	1.403 (6)
C3—C4	1.408 (5)	C23—H23	0.9300
C3—H3	0.9300	C24—C25	1.385 (5)
C4—C5	1.367 (5)	C24—H24	0.9300
C4—H4	0.9300	C25—C26	1.399 (5)
C5—C6	1.401 (5)	C25—H25	0.9300
C5—H5	0.9300	C26—N4	1.388 (4)
C6—N2	1.387 (4)	C27—N4	1.335 (4)
C7—N2	1.327 (4)	C27—N3	1.368 (4)
C7—N1	1.365 (4)	C27—C28	1.472 (5)
C7—C8	1.480 (5)	C28—C33	1.398 (5)
C8—C13	1.399 (5)	C28—C29	1.412 (5)
C8—C9	1.402 (5)	C29—O2	1.370 (4)
C9—O1	1.365 (4)	C29—C30	1.386 (5)
C9—C10	1.390 (5)	C30—C31	1.376 (5)
C10—C11	1.384 (5)	C30—H30	0.9300
C10—H10	0.9300	C31—C32	1.381 (6)
C11—C12	1.379 (5)	C31—H31	0.9300
C11—H11	0.9300	C32—C33	1.382 (5)
C12—C13	1.389 (5)	C32—H32	0.9300
C12—H12	0.9300	C33—H33	0.9300
C13—H13	0.9300	C34—O2	1.447 (4)
C14—O1	1.431 (4)	C34—C35	1.514 (5)
C14—C15	1.517 (5)	C34—H34A	0.9700
C14—H14A	0.9700	C34—H34B	0.9700
C14—H14B	0.9700	C35—C36	1.388 (5)
C15—C16	1.382 (5)	C35—C40	1.401 (5)
C15—C20	1.388 (5)	C36—C37	1.389 (6)
C16—C17	1.389 (5)	C36—H36	0.9300
C16—H16	0.9300	C37—C38	1.387 (6)
C17—C18	1.395 (5)	C37—H37	0.9300
C17—H17	0.9300	C38—C39	1.370 (6)
C18—C19	1.378 (6)	C38—H38	0.9300
C18—H18	0.9300	C39—C40	1.390 (6)
C19—C20	1.381 (5)	C39—H39	0.9300
C19—H19	0.9300	C40—H40	0.9300
C20—H20	0.9300	N1—H1	0.89 (4)
C21—N3	1.382 (5)	N3—H3A	0.83 (4)
			
N1—C1—C2	132.0 (3)	C21—C22—H22	121.8
N1—C1—C6	105.3 (3)	C22—C23—C24	121.7 (4)
C2—C1—C6	122.6 (3)	C22—C23—H23	119.2
C3—C2—C1	116.4 (4)	C24—C23—H23	119.2
C3—C2—H2	121.8	C25—C24—C23	121.5 (4)
C1—C2—H2	121.8	C25—C24—H24	119.2
C2—C3—C4	121.4 (4)	C23—C24—H24	119.2
C2—C3—H3	119.3	C24—C25—C26	117.5 (3)
C4—C3—H3	119.3	C24—C25—H25	121.2
C5—C4—C3	121.8 (3)	C26—C25—H25	121.2
C5—C4—H4	119.1	N4—C26—C21	110.2 (3)
C3—C4—H4	119.1	N4—C26—C25	129.5 (3)
C4—C5—C6	118.1 (3)	C21—C26—C25	120.3 (3)
C4—C5—H5	121.0	N4—C27—N3	112.3 (3)
C6—C5—H5	121.0	N4—C27—C28	122.0 (3)
N2—C6—C1	109.9 (3)	N3—C27—C28	125.6 (3)
N2—C6—C5	130.5 (3)	C33—C28—C29	118.6 (4)
C1—C6—C5	119.6 (3)	C33—C28—C27	117.9 (3)
N2—C7—N1	112.4 (3)	C29—C28—C27	123.3 (3)
N2—C7—C8	122.6 (3)	O2—C29—C30	124.4 (3)
N1—C7—C8	125.0 (3)	O2—C29—C28	115.7 (3)
C13—C8—C9	118.7 (3)	C30—C29—C28	119.9 (4)
C13—C8—C7	117.8 (3)	C31—C30—C29	120.2 (4)
C9—C8—C7	123.5 (3)	C31—C30—H30	119.9
O1—C9—C10	123.7 (3)	C29—C30—H30	119.9
O1—C9—C8	116.3 (3)	C30—C31—C32	120.8 (4)
C10—C9—C8	120.0 (3)	C30—C31—H31	119.6
C11—C10—C9	119.9 (3)	C32—C31—H31	119.6
C11—C10—H10	120.1	C31—C32—C33	119.8 (4)
C9—C10—H10	120.1	C31—C32—H32	120.1
C12—C11—C10	121.3 (3)	C33—C32—H32	120.1
C12—C11—H11	119.3	C32—C33—C28	120.7 (4)
C10—C11—H11	119.3	C32—C33—H33	119.7
C11—C12—C13	118.8 (4)	C28—C33—H33	119.7
C11—C12—H12	120.6	O2—C34—C35	113.5 (3)
C13—C12—H12	120.6	O2—C34—H34A	108.9
C12—C13—C8	121.3 (3)	C35—C34—H34A	108.9
C12—C13—H13	119.4	O2—C34—H34B	108.9
C8—C13—H13	119.4	C35—C34—H34B	108.9
O1—C14—C15	113.7 (3)	H34A—C34—H34B	107.7
O1—C14—H14A	108.8	C36—C35—C40	119.1 (4)
C15—C14—H14A	108.8	C36—C35—C34	122.3 (3)
O1—C14—H14B	108.8	C40—C35—C34	118.6 (3)
C15—C14—H14B	108.8	C35—C36—C37	120.3 (4)
H14A—C14—H14B	107.7	C35—C36—H36	119.9
C16—C15—C20	119.5 (4)	C37—C36—H36	119.9
C16—C15—C14	117.7 (3)	C38—C37—C36	120.4 (4)
C20—C15—C14	122.8 (3)	C38—C37—H37	119.8
C15—C16—C17	120.5 (3)	C36—C37—H37	119.8
C15—C16—H16	119.7	C39—C38—C37	119.6 (4)
C17—C16—H16	119.7	C39—C38—H38	120.2
C16—C17—C18	120.0 (4)	C37—C38—H38	120.2
C16—C17—H17	120.0	C38—C39—C40	121.0 (4)
C18—C17—H17	120.0	C38—C39—H39	119.5
C19—C18—C17	118.9 (4)	C40—C39—H39	119.5
C19—C18—H18	120.6	C39—C40—C35	119.7 (4)
C17—C18—H18	120.6	C39—C40—H40	120.1
C18—C19—C20	121.3 (4)	C35—C40—H40	120.1
C18—C19—H19	119.3	C7—N1—C1	107.1 (3)
C20—C19—H19	119.3	C7—N1—H1	131 (2)
C19—C20—C15	119.8 (4)	C1—N1—H1	121 (2)
C19—C20—H20	120.1	C7—N2—C6	105.3 (3)
C15—C20—H20	120.1	C27—N3—C21	107.0 (3)
N3—C21—C26	105.6 (3)	C27—N3—H3A	128 (3)
N3—C21—C22	131.8 (3)	C21—N3—H3A	125 (3)
C26—C21—C22	122.5 (3)	C27—N4—C26	104.9 (3)
C23—C22—C21	116.5 (3)	C9—O1—C14	119.4 (3)
C23—C22—H22	121.8	C29—O2—C34	117.9 (3)
			
N1—C1—C2—C3	−177.8 (4)	N4—C27—C28—C33	12.9 (5)
C6—C1—C2—C3	2.5 (5)	N3—C27—C28—C33	−170.4 (3)
C1—C2—C3—C4	−2.4 (5)	N4—C27—C28—C29	−163.0 (3)
C2—C3—C4—C5	1.4 (6)	N3—C27—C28—C29	13.7 (5)
C3—C4—C5—C6	−0.3 (5)	C33—C28—C29—O2	178.5 (3)
N1—C1—C6—N2	0.5 (4)	C27—C28—C29—O2	−5.7 (5)
C2—C1—C6—N2	−179.7 (3)	C33—C28—C29—C30	−2.3 (5)
N1—C1—C6—C5	178.7 (3)	C27—C28—C29—C30	173.6 (3)
C2—C1—C6—C5	−1.5 (5)	O2—C29—C30—C31	180.0 (4)
C4—C5—C6—N2	178.2 (4)	C28—C29—C30—C31	0.8 (5)
C4—C5—C6—C1	0.4 (5)	C29—C30—C31—C32	1.3 (6)
N2—C7—C8—C13	9.5 (5)	C30—C31—C32—C33	−1.8 (6)
N1—C7—C8—C13	−172.1 (3)	C31—C32—C33—C28	0.2 (6)
N2—C7—C8—C9	−167.8 (3)	C29—C28—C33—C32	1.8 (5)
N1—C7—C8—C9	10.6 (5)	C27—C28—C33—C32	−174.3 (3)
C13—C8—C9—O1	177.0 (3)	O2—C34—C35—C36	26.2 (5)
C7—C8—C9—O1	−5.7 (5)	O2—C34—C35—C40	−157.3 (3)
C13—C8—C9—C10	−2.5 (5)	C40—C35—C36—C37	0.6 (6)
C7—C8—C9—C10	174.8 (3)	C34—C35—C36—C37	177.1 (4)
O1—C9—C10—C11	−177.9 (3)	C35—C36—C37—C38	−0.1 (6)
C8—C9—C10—C11	1.6 (5)	C36—C37—C38—C39	−0.7 (6)
C9—C10—C11—C12	0.5 (6)	C37—C38—C39—C40	1.0 (6)
C10—C11—C12—C13	−1.5 (6)	C38—C39—C40—C35	−0.4 (6)
C11—C12—C13—C8	0.5 (6)	C36—C35—C40—C39	−0.4 (5)
C9—C8—C13—C12	1.5 (5)	C34—C35—C40—C39	−176.9 (3)
C7—C8—C13—C12	−176.0 (3)	N2—C7—N1—C1	1.5 (4)
O1—C14—C15—C16	169.2 (3)	C8—C7—N1—C1	−177.0 (3)
O1—C14—C15—C20	−11.9 (5)	C2—C1—N1—C7	179.1 (4)
C20—C15—C16—C17	0.5 (5)	C6—C1—N1—C7	−1.2 (4)
C14—C15—C16—C17	179.4 (3)	N1—C7—N2—C6	−1.2 (4)
C15—C16—C17—C18	−0.6 (5)	C8—C7—N2—C6	177.4 (3)
C16—C17—C18—C19	0.0 (5)	C1—C6—N2—C7	0.4 (4)
C17—C18—C19—C20	0.6 (6)	C5—C6—N2—C7	−177.6 (4)
C18—C19—C20—C15	−0.7 (5)	N4—C27—N3—C21	0.1 (4)
C16—C15—C20—C19	0.2 (5)	C28—C27—N3—C21	−176.8 (3)
C14—C15—C20—C19	−178.7 (3)	C26—C21—N3—C27	0.4 (4)
N3—C21—C22—C23	−176.6 (4)	C22—C21—N3—C27	176.7 (4)
C26—C21—C22—C23	−0.9 (5)	N3—C27—N4—C26	−0.6 (4)
C21—C22—C23—C24	0.8 (6)	C28—C27—N4—C26	176.5 (3)
C22—C23—C24—C25	−1.3 (6)	C21—C26—N4—C27	0.9 (4)
C23—C24—C25—C26	1.7 (5)	C25—C26—N4—C27	−177.9 (4)
N3—C21—C26—N4	−0.8 (4)	C10—C9—O1—C14	−1.1 (5)
C22—C21—C26—N4	−177.6 (3)	C8—C9—O1—C14	179.4 (3)
N3—C21—C26—C25	178.1 (3)	C15—C14—O1—C9	−78.0 (4)
C22—C21—C26—C25	1.4 (5)	C30—C29—O2—C34	−5.5 (5)
C24—C25—C26—N4	176.9 (3)	C28—C29—O2—C34	173.7 (3)
C24—C25—C26—C21	−1.7 (5)	C35—C34—O2—C29	80.6 (4)

### Hydrogen-bond geometry (Å, °)

**Table:**

*D*—H···*A*	*D*—H	H···*A*	*D*···*A*	*D*—H···*A*
N1—H1···O1	0.89 (4)	2.20 (3)	2.667 (4)	112 (3)
N1—H1···N4^i^	0.89 (4)	2.18 (3)	3.008 (4)	154 (3)
N3—H3A···O2	0.83 (4)	2.20 (5)	2.670 (4)	117 (4)
N3—H3A···N2^ii^	0.83 (4)	2.18 (5)	2.918 (4)	148 (4)
C14—H14A···Cg1^i^	0.97	2.88	3.736 (4)	148
C14—H14B···Cg4^iii^	0.97	2.92	3.721 (4)	141
Cg3—···.Cg2^i^	.	.	3.859 (2)	.

Symmetry codes: (i) *x*−1, −*y*+1, *z*−1/2; (ii) *x*+1/2, −*y*+1/2, *z*+1/2; (iii) *x*−1, *y*, *z*−1.
